# Neuroprotection in late life attention-deficit/hyperactivity disorder: A review of pharmacotherapy and phenotype across the lifespan

**DOI:** 10.3389/fnhum.2022.938501

**Published:** 2022-09-26

**Authors:** Cintya Nirvana Dutta, Leonardo Christov-Moore, Hernando Ombao, Pamela K. Douglas

**Affiliations:** ^1^Biostatistics Group, Computer, Electrical and Mathematical Sciences and Engineering Division, King Abdullah University of Science and Technology, Thuwal, Saudi Arabia; ^2^School of Modeling, Simulation, and Training, and Computer Science, University of Central Florida, Orlando, FL, United States; ^3^Brain and Creativity Institute, University of Southern California, Los Angeles, CA, United States; ^4^Department of Psychiatry and Biobehavioral Medicine, University of California, Los Angeles, Los Angeles, CA, United States

**Keywords:** ADHD, MRI, psychostimulants, cognitive impairment, neurodegeneration

## Abstract

For decades, psychostimulants have been the gold standard pharmaceutical treatment for attention-deficit/hyperactivity disorder (ADHD). In the United States, an astounding 9% of all boys and 4% of girls will be prescribed stimulant drugs at some point during their childhood. Recent meta-analyses have revealed that individuals with ADHD have reduced brain volume loss later in life (>60 y.o.) compared to the normal aging brain, which suggests that either ADHD or its treatment may be neuroprotective. Crucially, these neuroprotective effects were significant in brain regions (e.g., hippocampus, amygdala) where severe volume loss is linked to cognitive impairment and Alzheimer’s disease. Historically, the ADHD diagnosis and its pharmacotherapy came about nearly simultaneously, making it difficult to evaluate their effects in isolation. Certain evidence suggests that psychostimulants may normalize structural brain changes typically observed in the ADHD brain. If ADHD itself is neuroprotective, perhaps exercising the brain, then psychostimulants may not be recommended across the lifespan. Alternatively, if stimulant drugs are neuroprotective, then this class of medications may warrant further investigation for their therapeutic effects. Here, we take a bottom-up holistic approach to review the psychopharmacology of ADHD in the context of recent models of attention. We suggest that future studies are greatly needed to better appreciate the interactions amongst an ADHD diagnosis, stimulant treatment across the lifespan, and structure-function alterations in the aging brain.

## Introduction

Attention-deficit/hyperactivity disorder is one of the most common psychiatric disorders in children (Biederman and Faraone, 2005; Simonoff et al., 2008; Banaschewski et al., 2017; Sayal et al., 2018). The psychiatric nosology of ADHD is primarily based on its clinical phenomenology and diagnosed with the use of standardized diagnostic rating scales (Thome et al., 2012). Classically, ADHD has been described as a highly heritable (60–75%) neurodevelopmental disorder, characterized along the domains of inattention, hyperactivity, and impulsivity (Wolraich et al., 1996; Swanson et al., 1998) with increased novelty seeking behavior recently noted to be a core feature of ADHD (Donfrancesco et al., 2015).

One of the defining characteristics of ADHD is the heterogeneity of behavioral presentations, yet the diagnosis replicates with reasonable success rates across practitioners (McGough, 2014; Faraone et al., 2015). Early descriptions of this disorder suggested that hyperkinetic and inattentive symptoms during childhood generally attenuated by late adolescence (Laufer and Denhoff, 1957), a description maintained through the DSM-IV. However, it is now recognized that 60–70% of pediatric cases continue to have functional impairments into adulthood (Fischer et al., 2002; Sibley et al., 2016), though in many cases only three to four symptoms (sub-threshold for diagnostic criteria) may continue after adolescence (Faraone et al., 2006).

It is evident that variations in the epidemiology and pharmacoepidemiology of ADHD are found across the globe. A meta-analysis of worldwide prevalence in 27 countries in children and adolescents with ADHD was reported at 3.4% (Confidence Interval 95% 2.6–4.5) (Polanczyk et al., 2015). For example, it was estimated that the prevalence rate of childhood ADHD in 2020 would be 1% (95% Confidence Interval: 0.875–1.125) in Slovenia (Štuhec et al., 2015), and less than 50% of patients in Slovenia were treated with medication for pediatric ADHD (Stuhec and Locatelli, 2017). In contrast, a systematic and meta-analysis report in China found a childhood ADHD prevalence rate of 6.26% (95% Confidence Interval: 5.36–7.22%) (Wang et al., 2017) and utilization of prescriptions for ADHD medication was more than 50% in patients aged 6–11 years (Wang et al., 2021). Variability of prevalence rates could be explained by methodological approaches, specifically in diagnostic criteria, source of information, and requirement of impairment for diagnosis (Polanczyk et al., 2014; Visser et al., 2014). Pharmacoepidemiology studies have revealed geographic variability both globally (Raman et al., 2018) and cross-nationally (Zito, 2000) with respect to diagnostic practices, clinical management, and societal differences toward pharmacotherapy in children. Differences in clinical decision-making and medication utilization are also time-varying, further complicating lifespan studies on ADHD populations (Chang et al., 2019).

A number of studies have examined the trajectory and stability of ADHD symptoms across the lifespan. In children, approximately 5% (predominantly male) prevalence rates are generally reported (e.g., Wolraich et al., 1996). In contrast, adult prevalence rates are more gender balanced and estimated at ∼3% (Fayyad et al., 2007). However, adult prevalence rates are higher if the DSM-5 requirement for a childhood-onset (before age 12) is removed (Caye et al., 2016). A recent meta-analysis reported 2.58% prevalence for adult ADHD with a childhood onset, with 6.76% of adults overall having ADHD symptoms (Song et al., 2021). A longitudinal study across four decades found that 90% of adult ADHD cases lacked a childhood history (Moffitt et al., 2015).

Taken together, these reports suggest that there may be at least two different disease trajectories, and the potential for the existence of multiple etiologies (Moffitt et al., 2015; Caye et al., 2016). There is some evidence that the neural substrates subtending the inattentive phenotype are distinct from other ADHD presentations. For example, when independent component analysis, a technique used to isolate statistically independent brain networks, was applied to resting state fMRI data, approximately double the number of components were computed in inattentive subjects compared to either typically developing or hyperactive ADHD subjects (Colby et al., 2012).

Despite copious research, a clear link between the clinical features of ADHD and its biological substrates remains at least somewhat elusive. However, neuropsychological, imaging and genetic studies have converged on a few central features with correlates across domains. Contemporary neurocognitive models of ADHD consider deficits in executive functioning, specifically response disinhibition, to be a core deficit (Curatolo et al., 2010; Purper-Ouakil et al., 2011). Neuropsychological and imaging studies have implicated abnormalities in prefrontal cortex (PFC), a brain region thought to be critical for many aspects of executive function such as sustaining and dividing attention as well as inhibiting distraction (Arnsten and Li, 2005; Seidman, 2006; Christakou et al., 2013). PFC lesions often produce a behavioral profile of distractibility, forgetfulness, impulsivity, poor planning, and locomotor hyperactivity similar to ADHD (Brennan and Arnsten, 2010).

Attention-deficit/hyperactivity disorder is mitigated by pharmacological medications that increase concentrations or residence time of dopamine and norepinephrine in the synapse. According to evidence-based guidelines and meta-analyses of pharmacological management of ADHD, stimulants are considered the first-line treatment in both children (Faraone and Buitelaar, 2010; Chan et al., 2016; Catalá-López et al., 2017; Wolraich et al., 2019) and adults (Rostain, 2008; Cunill et al., 2016; De Crescenzo et al., 2017; Stuhec et al., 2019), while non-stimulants remain second-line option treatments (Bolea-Alamañac et al., 2014; Chan et al., 2016; Caye et al., 2019).

Here, we suggest that more studies should focus on examining pharmacotherapy-based treatment for ADHD across the lifespan. However, isolating the effects of psychostimulant treatment is difficult for a number of reasons. First, the high rate of psychiatric comorbidity in adults (Kessler, 2006; Sobanski et al., 2007) and children (Kadesjö and Gillberg, 2001; Reale et al., 2017) with ADHD frequently necessitates combination of psychopharmacology, which may pose a risk of drug–drug interactions, and further complicate lifespan studies of pharmacotherapy. This is important in light of the differences in profiles of dopaminergic capacity and dopamine transporter availability in ADHD and comorbidities (Howes et al., 2012; Jauhar et al., 2017). Second, psychostimulants are known to carry the risk of abuse (Barkley et al., 2003; Weaver et al., 2005), which is pertinent given that youths with ADHD are more likely to experiment with psychoactive substances (Elkins et al., 2007; Wilens et al., 2011), and its increasing usage (Zuvekas and Vitiello, 2012; Olfson et al., 2013) may further complicate studies on psychostimulant treatment alone. Growing evidence also suggests an overlap in genetic susceptibility between substance abuse and ADHD (Goldman et al., 2005; Lesch et al., 2008; Wang, 2013; Demontis et al., 2019). Current guidelines recommend the non-stimulant atomoxetine as a first-choice treatment for adults with substance use disorder (SUD) and ADHD, but evidence of efficacy is still lacking (Kooij et al., 2010). Although evidence on the use of stimulants for patients with SUD and ADHD is mixed, Consensus Statements recommend the use of stimulants in adults (Klassen et al., 2012; Torgersen et al., 2013) and adolescents (Özgen et al., 2020). Therefore, there is a need to clarify if substance abuse patients with ADHD will abuse prescription drugs and if treatments will maintain their effectiveness in the presence of SUD (Mariani and Levin, 2007; Molina et al., 2013; Kooij et al., 2019; Wilens et al., 2003; MTA Cooperative Group, 2004). Third, there is still a need for guidelines on evidence-based hierarchies on the efficacy and tolerability of all pharmacological treatments for ADHD in children and adults. Evidence from a systematic and network meta-analysis study supports methylphenidate in children and adolescents, and amphetamines in adults, as the preferred first-choice medications for short-term treatment, but new research should assess long-term effects of these drugs (Cortese et al., 2018). On the other hand, methylphenidate was recommended as the first-line treatment for ADHD in adults in the UK (Bolea-Alamañac et al., 2014), and statistically significant response of lisdexamfetamine was found in children and adolescents with ADHD (Roskell et al., 2014) but safety data proved inconclusive due to low event rates. Fourth, genes (Bruxel et al., 2014, 2015; Myer et al., 2018) and gene-environment (Pagerols et al., 2018) factors influence the efficacy of pharmaceutical drugs for ADHD (Elsayed et al., 2020). Fifth, only stimulants and atomoxetine were found to reach Ia evidence levels (Bolea-Alamañac et al., 2014), thus requiring further evidence-based medicine research on other common and novel pharmacological treatments for individuals with ADHD of all ages.

**FIGURE 1:**
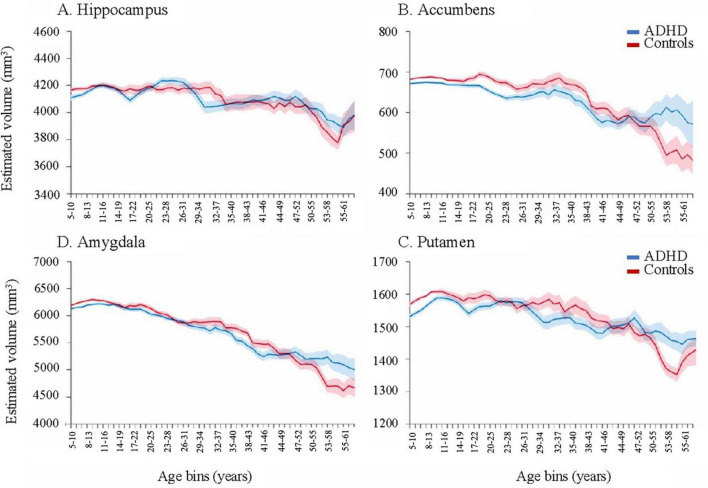
Moving averages, corrected for age, sex, intracranial volume, and site for subcortical volumes. Error bars denote the standard error. As individuals with ADHD approach late adulthood (>60), volume remains relatively higher than controls in certain subcortical structures, such as the hippocampus, accumbens, putamen, and amygdala. Reproduced with permission (Hoogman et al., 2017).

Lastly, and most importantly, a delay in brain degeneration was observed in adults with ADHD regardless of medication status. Hoogman et al. (2017) found that in certain brain regions, age-related volumetric decline (age > 60) was less pronounced in the ADHD group compared to age-matched controls, notably in amygdala and hippocampal regions (Figure 1). Given that volumetric loss in these brain regions is a hallmark of cognitive impairment later in life, there is a clear need for studies that examine the relationships between neurodegeneration, ADHD, psychostimulant use, and their neuroimaging-genetic correlates.

Given these concerns, it is critically important to review how psychostimulant treatment for ADHD affects the structural integrity of the brain throughout the lifespan and how these treatments should best be administered to patients with ADHD, given that medication may resolve differences in the ADHD brain, and thus counteract the potential for ADHD itself to provide neuroprotection in late life. Bridging multiple levels of description from a molecular to a system levels of abstraction may improve our understanding of how these substances enhance or attenuate behavioral symptoms across ADHD presentations. It may also lead to quantitative metrics that aid in individualized treatment regimens during early and late stage titration periods across the lifespan.

In this paper, we first review the pharmacology of drugs commonly prescribed to children and adults with ADHD. We then discuss structural and functional neuroimaging differences in the ADHD brain. Interestingly, there is mixed evidence as to whether or not pharmaceutical treatment normalizes these structural differences. Here, we review this evidence in the context of recent findings which note a delay in late life neurodegeneration in the ADHD population, particularly in brain regions noted to decline in Alzheimer’s disease (AD). An important future line of inquiry may address the overlap or lack thereof between ADHD, neurodegenerative diseases, and their respective genetics. Finally, we suggest that improved models of ADHD and pharmaceutical treatment across the lifespan may help update clinical decision-making in light of recent evidence. Our method for identifying relevant literature for inclusion in this review is described in Supplementary Methods.

### Principal drug targets: Catecholaminergic systems

One of the defining characteristics of ADHD is its heterogeneity of symptom presentation, and multiple genetic variations have been linked to increased risk for the ADHD (Faraone and Mick, 2010). A recent review found that about a third of ADHD’s heritability is due to polygenetic components, each contributing small effects to the overall presentation (Faraone and Larsson, 2019). In this sense, multiple genetic variants and combinations thereof may subtend a given cluster of behavioral attributes grouped under a given ADHD behavioral phenotype, thus creating an equifinality effect. Conversely, identical twin studies have demonstrated that a single genotype can lead to a diversity of presentations and a spectrum of severities. Thus, both equifinality and multifinality effects are evident with ADHD. A variety of neurocognitive endophenotypes that mediate pathways between the genotype and phenotype have been identified and have helped unravel the complexity of this disease across the lifespan (Boxhoorn et al., 2019).

Several genetic markers of ADHD identified to date involve alterations in dopamine (DA) and noradrenaline signaling (NA) (Pliszka et al., 1996). There are certain similarities between dopaminergic and noradrenergic signaling and disruptions thereof in the brain. For example, both DA and NA are primarily synthesized in localized nuclei: the substantia nigra and locus coeruleus, respectively (Beaulieu and Gainetdinov, 2011; Bhatia and Saadabadi, 2020). However, both the dopaminergic (Friston et al., 1994) and noradrenergic (Smythies, 2005) systems are thought to project diffusely across the brain (Stahl, 2003; Gerfen and Surmeier, 2011; Purves, 2012), thus having the potential to “broadcast” their responses to exert widespread effects in the brain. Both systems also exert varied effects across different time scales depending on whether response patterns are (sustained) or phasic (transient) and there is evidence that both exert varied effects depending on whether they modulate top-down or bottom-up processing (Manev and Uz, 2009; Badgaiyan et al., 2015). Noradrenaline is thought to modulate multiple functions in the brain from learning rates (Aston-Jones and Cohen, 2005; Sales et al., 2019) to attention (Sved et al., 2001). Tonic firing of the locus coeruleus is correlated with arousal levels and behavioral flexibility (Mefford and Potter, 1989). In contrast, salient stimuli are thought to evoke more brief, high frequency phasic responses (Sales et al., 2019).

Therefore, there are relatively few candidate pathways that are thought to be involved in the ADHD phenotype, in contrast to many other psychiatric disorders (Hauser et al., 2016). Here, we summarize the major classes of pharmaceuticals aimed at treating ADHD and their putative involvement with DA and NA signaling.

#### Stimulants

Stimulants are considered the gold standard pharmaceutical treatment and generally recommended as a first line of treatment for children and adults with severe or moderate ADHD (Centers for Disease Control and Prevention [CDC], 2005; Pliszka and AACAP Work Group on Quality Issues, 2007; Subcommittee on Attention-Deficit/Hyperactivity Disorder, Steering Committee on Quality Improvement and Management, Wolraich et al., 2011; Bolea-Alamañac et al., 2014). An estimated 70% of patients respond favorably to the initial stimulant selection (Elia et al., 1991; Schachter et al., 2001; Barbaresi et al., 2006). Methylphenidate and amphetamine treatment for ADHD reach level Ia in terms of effectiveness (Bolea-Alamañac et al., 2014) and as revealed by meta-analyses comparing stimulant drug and placebo interventions in both children (Faraone and Buitelaar, 2010; Cortese et al., 2018) and adults (De Crescenzo et al., 2017; Cortese et al., 2018) with ADHD. However, all stimulant formulations of methylphenidate (MPH) and amphetamine (AMPH) are classified as Schedule II drugs, denoting high risk for misuse, diversion, and potential neurotoxicity (Wilens et al., 2003, 2008; Casey et al., 2007; Clemow and Walker, 2014; McGough, 2014), as well as tolerance or sensitization if used at high doses. In the case of risk of misuse of psychostimulants or comorbidity to SUD, atomoxetine is recommended as a first-choice treatment, however, evidence of efficacy is limited (Bolea-Alamañac et al., 2014). Here, we briefly summarize the neuropharmacology and cellular pathway alterations induced *via* these key stimulant drugs while also noting its long-term effects.

##### Amphetamine

Amphetamine (AMPH; IUPAC name: 1-phenylpropane-2-amine), the active component of drugs like Adderall, Dexedrine, and Vyvanse, acts as an indirect agonist to catecholamines. The term amphetamine typically refers to the racemic mixture, or equal parts mixture, of dextro and levo amphetamine, though the drugs themselves typically have varying mixtures of these enantiomers, as well as derivations thereof. The cellular signaling pathways altered by AMPH are complex. Repeated exposure to psychostimulant drugs can lead to both acute and enduring effects on neurobiological substrates in the brain (Howell and Kimmel, 2008). The primary short-term effects of these drugs tend to increase the concentration and/or residence time of monoamines [e.g., dopamine (DA), norepinephrine (NE)] in the synapse by promoting efflux into the synaptic cleft, and inhibiting or competing with monoamines for reuptake.

**FIGURE 2:**
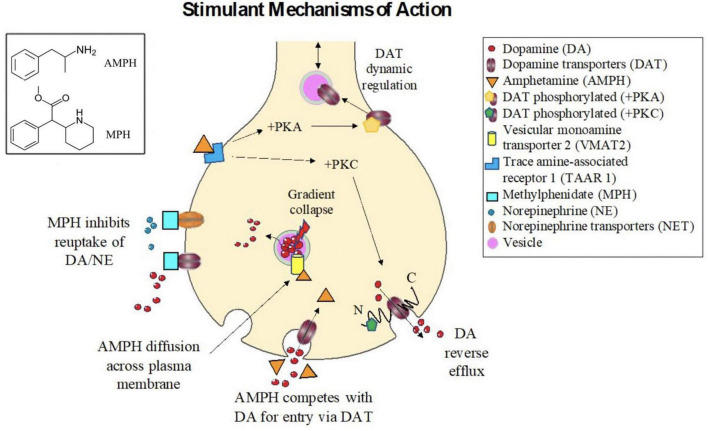
Mechanisms of action for stimulant drugs. (Box, **Upper Left**). Chemical structures for the **(top)** Amphetamine and **(bottom)** Methylphenidate. Stimulant mechanisms of action. Methylphenidate (MPH) acts as an antagonist drug by binding and inhibiting dopamine transporters (DATs) and norepinephrine transporters (NETs), thereby reducing reuptake of (DA) and norepinephrine (NE) from the synaptic cleft. Amphetamine (AMPH) can enter neurons either *via* direct diffusion across the plasma membrane or *via* competition with the endogenous transport machinery for DA (i.e., the dopamine transporter (DAT)). Once inside the cell, AMPH binds at distinct sites on the vesicular monoamine transporter 2 (VMAT2), causing vesicular gradient collapse, which in turn induces DA release from the vesicles into the cytosol, increasing intracellular concentrations of DA, and reversing the concentration gradient across the cellular membrane, promoting efflux of DA into the synapse. AMPH is also strong trace amine associated receptor 1 (TAAR1) agonist. Activation of TAAR1, through protein kinase A (PKA), protein kinase C (PKC), and adenylyl cyclase signaling, causes phosphorylation of DAT, which has long N and C intracellular termini. Phosphorylation of the N-terminus can promote DA reverse efflux into the synapse. Phosphorlylation of DAT can also inhibit its function, thus diminishing the plasma membrane transporter from clearing DA from the synapse. The number of DATs localized to the plasma membrane is also dynamically regulated, and a reduction of DATs following AMPH treatment is thought to subtend many of the long-term effects of stimulant treatment.

Amphetamine is lipophilic, and can therefore enter neurons *via* direct diffusion across the cellular membrane (Gulaboski et al., 2007). However, the chemical structure of AMPH is similar to DA (Figure 2); it is therefore unsurprising that AMPH can also enter *via* these monoamine transporters (Mortensen and Amara, 2003; Sulzer et al., 2005; Howell and Kimmel, 2008) and compete with these endogenous ligands for their transport machinery (Mortensen and Amara, 2003; Zhu and Reith, 2008) thereby reducing monoamine clearance from the synapse. For example, the dopamine transporter (DAT), a monoamine transporter, is an integral transmembrane protein that typically functions to import or clear DA from the synapse, and represents an important target site for both MPH and AMPH (Spencer et al., 2005; Larisch et al., 2006).

Amphetamine can also increase efflux of monoamines into the synapse *via* multiple mechanisms. First AMPH can inhibit of a variety of vesicular monoamine transporters (VMATs), including VMAT1 and VMAT2, as well as interacting with excitatory amino acid transporters (e.g., SLC1A1), which are also biological substrate targets for non-stimulant drugs (e.g., atomoxetine) (Hammerness et al., 2009). In particular, VMAT2 is a transporter protein, which serves to import monoamines from the cytosol into vesicles for storage and later release into the synapse. AMPH can bind to VMAT2 and inhibit its normal vesicular import function, leading to a reduction of monoamines released during exocytosis. However, when AMPH enters synaptic vesicles expressing VMAT2, the vesicular pH gradient collapses, which in turn causes stored amines to be released into the cytosol, thus increasing their intracellular concentration (Sulzer and Rayport, 1990; Sulzer et al., 2005). The increased cytosolic concentration of monoamines, in turn, induces “reverse transport” through primary plasma membrane monoamine transporters (Sulzer et al., 1995; John and Jones, 2007) such as DAT. DAT is an Na+/Cl– symporter (Khelashvili et al., 2015), driven primarily *via* Na+ ion and amine concentration gradients (Wheeler et al., 1993; Bröer and Gether, 2012). Therefore, reverse efflux is more likely during neuronal depolarization.

Amphetamine is also a powerful agonist for trace amine-associated receptor 1 (TAAR1), a G-protein coupled receptor, which acts in conjunction with plasma membrane monoamine transporters to regulate the extracellular concentrations of these monoamine neurotransmitters (Kobayashi et al., 2010). By activating TAAR1, protein kinase A (PKA) and protein kinase C (PKC) are activated leading to increased cyclic AMP, which in turn inhibits the synaptic influx function of integral membrane monoamine transporters, again resulting in diminished clearance from the synapse (Kebabian, 1978; Johnson et al., 2005a; Miller, 2011). The short term intracellular mechanisms of action of AMPH: increasing monoamine release into the synapse, and diminish reuptake rates, are summarized in Figure 2.

Differential functional brain activities are present in dexamphetamine (*S*-amphetamine, D-amphetamine) and levoamphetamine (*R*-amphetamine, lisdexamfetamine). Presently, the modern formulation of amphetamine for ADHD treatment is a combination of racemic amphetamine and d-amphetamine (Knackstedt, 2013). Lisdexamfetamine is metabolized by red blood cells to yield its active metabolite, D-amphetamine, and L-lysine (Pennick, 2010). D-amphetamine was shown to be 3–4 times more potent than *l*-amphetamine in the striatum, but not in the cortex, thus, D-amphetamine has a greater affinity for striatal DAT than *l*-amphetamine (Knackstedt, 2013). *S*-Amphetamine is more potent in CNS stimulation, while *R*-amphetamine is slightly more potent in its cardiovascular action (Varesio and Veuthey, 1995). Similar to dexamphetamine, lisdexamfetamine is posed to be an effective treatment of ADHD (evidence level Ia) in randomized, double-blinded, placebo-controlled trials in children (Biederman et al., 2007) and adults (Adler et al., 2008) with ADHD and in open-label long-term investigations in adults (Weisler et al., 2009). Side effect profiles are similar in both D-amphetamine and lisdexamfetamine, but recreational abuse may be substantially lower in lisdexamfetamine than that of immediate-release D-amphetamine (Jasinski and Krishnan, 2009).

##### Methylphenidate

Methylphenidate (MPH; IUPAC name: methyl 2-phenyl-2-piperidin-2-ylacetate), active in drugs such as Ritalin, Methylin, Concerta, and Focalin, blocks dopamine (DAT) and norepinephrine (NET) transporter (Kuczenski and Segal, 1997; Solanto, 1998; Heal and Pierce, 2006; Iversen, 2009), which leads to increased dopamine (DA) and norepinephrine (NE) concentrations and/or residence time within the synaptic cleft following impulse release (Volkow et al., 2001, 2005). MPH also shares part of its basic structure with catecholamines, which is putatively related to its antagonistic role as a DA/NE reuptake inhibitor (Figure 2). Systematic reviews and meta-analyses reveal differences in the efficacy profiles of amphetamine (AMPH) and methylphenidate (MPH) in adults and children with ADHD. In two meta-analyses, both AMPH and MPH showed comparable efficacy in adults (Faraone and Glatt, 2009) and children (Catalá-López et al., 2017) with ADHD. Other meta-analyses revealed amphetamine as having greater efficacy than methylphenidate and non-stimulants in children (Faraone and Buitelaar, 2010; Stuhec et al., 2015; Joseph et al., 2017) and adults (De Crescenzo et al., 2017; Stuhec et al., 2019) with ADHD. A reduction of adult ADHD symptoms was observed in lisdexamfetamine, whereas mixed amphetamine salts and methylphenidate reduced symptoms moderately compared to placebo (Stuhec et al., 2019). In children and adolescents with ADHD, efficacy in reduction of ADHD symptoms compared to placebo is small for bupropion, modest for atomoxetine and methylphenidate, and high efficacy for lisdexamfetamine (Stuhec et al., 2015). Similarly, lisdexamfetamine dimesylate had the highest efficacy than guanfacine extended release, atomoxetine, and methylphenidate extended release among children and adolescents with ADHD (Joseph et al., 2017). Tolerability of both treatments are relatively comparable (Faraone, 2018). It must be noted that current observations of methylphenidate treatment on dopaminergic signaling are based on the effects seen in normal versus pathological conditions. In healthy animals, there are long term changes in reduction of striatal dopamine transporters densities (Moll et al., 2001) and variability in firing rate among dopaminergic midbrain neurons (Brandon et al., 2003; Viggiano et al., 2003, 2004); although the neurotrophic effects may differ (Gill et al., 2012) based on age (Fukui et al., 2003) and quantity of dose (Volkow et al., 1998). In rat midbrain slices, methylphenidate inhibits dopamine transporters and this depression of firing is mediated by synthesized dopamine that increases intracellularly due to reuptake inhibition (Morón et al., 2002; Federici et al., 2005).

Converging evidence from *in vitro* to rodent to human studies suggest that the d enantiomer is thought to mediate therapeutic effects for the behavioral symptoms of ADHD (e.g., Patrick et al., 1987; Teo et al., 2003; Ding et al., 2004; Markowitz and Patrick, 2008; Markowitz et al., 2009), though clinical evidence suggests that the *l*-enantiomer is very effective at treating behavioral symptoms in children (Hubbard et al., 1989). Many studies suggest that the chirality is important and that the l enantiomer is non-specific, and may actually interfere with the specific targeting of the d version of this molecule.

##### Long term effects of stimulant treatment

The extent to which long-term exposure to stimulants induces persistent neuroplasticity has not been fully elucidated, partly due to the fact that an X-ray structure for DAT was only reported somewhat recently in Drosophila (Penmatsa et al., 2013). Phosphorylation of monoamine transporters induced *via* a range of kinases (e.g., protein kinase C) appears to alter transporter functionality (Johnson et al., 2005a). The DAT has long N and C intracellular termini, which provide multiple sites for phosphorylation and regulation (Foster et al., 2003; Giambalvo, 2003). For example, phosphorylation of the N-terminus of DAT appears to be tightly linked to DA efflux (e.g., Khoshbouei et al., 2004), and may be requisite for AMPH induced DA efflux (e.g., Kantor and Gnegy, 1998; Mortensen and Amara, 2003).

Stimulant treatment can also alter the quantity and plasmalemal expression of monoamine transporters (Krause et al., 2000; Kahlig and Galli, 2003; Johnson et al., 2005a; McGinty et al., 2008; Zhu and Reith, 2008). *In vitro* studies in rat striatal synaptosomes have demonstrated increased trafficking of DAT to the plasma membrane following rapid treatment (∼1 min) with AMPH (Furman et al., 2009). In contrast, numerous *in vitro* studies have demonstrated that longer exposure (>∼30 min) results in DAT internalization (e.g., Chi and Reith, 2003; Johnson et al., 2005b). In untreated adult humans with ADHD, SPECT imaging studies have shown an increase in DAT specific ligand binding (TRODAT-1) in striatum compared to controls, and a significant reduction in binding after 4 weeks of MPH treatment (Krause et al., 2000), consistent with longer exposure *in vitro* studies.

Overall, long-term effects of stimulant treatment may result from a number of factors including phosphorylation of monoamine transporters (Khelashvili et al., 2015), alterations in the dynamic expression and quantity of transporters at the plasma membrane (Zhu and Reith, 2008), and the induction of downstream gene expression (McGinty et al., 2008; Zhu and Reith, 2008). Alterations in brain structure and function revealed by neuroimaging may provide further insight into prolonged effects of pharmacotherapy for ADHD (see neuroimaging section below).

#### Non-stimulants

About 10–30% of ADHD patients respond poorly to stimulant medication (Greenhill et al., 2002; Spencer et al., 2004). For these patients, non-stimulant agents can be second-line treatment options, used when stimulants are ineffective, or used in combination with stimulants to enhance treatment response (Zito et al., 2008; Sallee et al., 2009; Subcommittee on Attention-Deficit/Hyperactivity Disorder, Steering Committee on Quality Improvement and Management, Wolraich et al., 2011; Kornfield et al., 2013). The two most common FDA-approved non-stimulants for ADHD are Alpha-2-Adrenergic Agonists and Atomoxetine.

##### Alpha 2-adrenergic agonists

**FIGURE 3:**
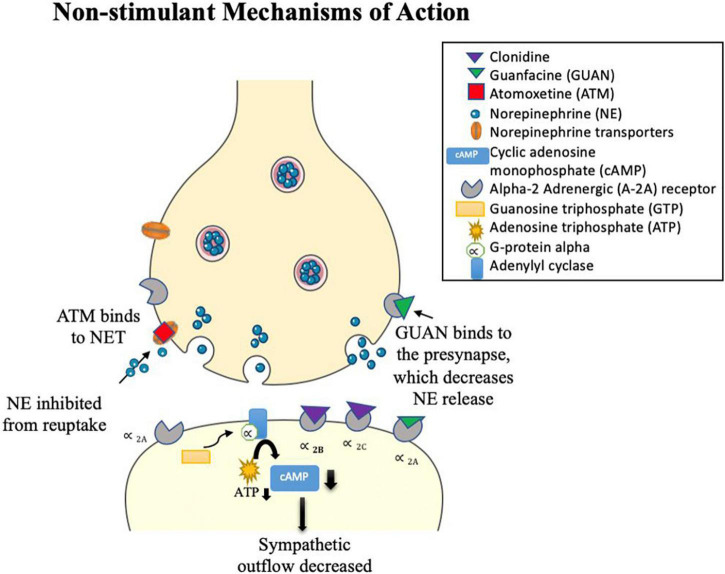
Clonidine and Guanfacine alpha 2 adrenergic agonist drugs. Clonidine acts on various α_2_- adrenergic subtypes (α_2A_, α_2B_, and α_2C_), while guanfacine is a selective α_2A_ adrenergic agonist. Presynaptic stimulation of these autoreceptors inhibits NE release. Post-synaptic stimulation of these α_2_-adrenergic receptors activates a series of steps: G-protein alpha is released and sent to adenylyl cyclase by guanosine triphosphate (GTP). Binding to adenylyl cyclase lowers adenosine triphosphate (ATP), which decreases the intracellular production of the secondary messenger cyclic adenosine monophosphate (cAMP). This reduces phosphorylation and inhibits NE release. Both drugs act as sympatholytic drugs, decreasing sympathetic outflow. Atomoxetine (ATM) is a selective antagonist drug to NE and has a lower affinity to serotonin (SERT) and dopamine (DAT) transporters. ATM blocks NETs, thereby increasing NE in the synaptic cleft.

Alpha 2-adrenergic agonists typically bind to α _2_ adrenergic receptors, causing vasodilation of the arteries (see Figure 3; Hossmann et al., 1980; Cinnamon Bidwell et al., 2010). Clonidine [brand names Kapvay and Catapres; IUPAC name: *N*-(2,6-dichlorophenyl)-4,5-dihydro-1H-imidazole-2-amine] is an imidazoline derivative and a non-selective α_2_ adrenergic agonist. It binds to all subtypes of α_2_-adrenergic receptors (Roth and Driscol, 2011): α_2A_, α_2B_, α_2C_. Alpha _2A_-receptors have been found in the locus coeruleus, frontal cortex, cerebellum, septum, hypothalamus, and hippocampus (Guyenet et al., 1994; Grijalba et al., 1996; Talley et al., 1996; Saunders and Limbird, 1999; Offermans and Rosenthal, 2004). Alpha _2B_-receptors are localized in the thalamus, hippocampus, and cerebellar Purkinje layer (Saunders and Limbird, 1999). Alpha _2C_-receptors are expressed in several subcortical regions including the thalamus, amygdala, substancia nigra and ventral tegmentum area (Saunders and Limbird, 1999; Offermans and Rosenthal, 2004). Guanfacine [brand names Intuniv, Estulic, and Tenex; IUPAC name: *N*-(diaminomethylidene)-2-(2,6 dichlorophenyl)acetamide] is a selective agonist drug for α_2A_ adrenergic receptors, with a 60x and 20x higher affinity to these receptors than to α_2B_- and α_2C_-receptors, respectively (Uhlén et al., 1995; Kawaura et al., 2014). Clonidine and guanfacine are level Ib in evidence-based medicine practice (Bolea-Alamañac et al., 2014), signifying level of evidence for individual RCTs (with narrow confidence intervals) (Burns et al., 2011). Combination of stimulants with alpha 2 agonists (clonidine, guanfacine) and monotherapy of alpha 2 agonists have not been extensively studied in adults (Bolea-Alamañac et al., 2014). Comorbidity of ADHD and other psychiatric conditions alter evidence levels to III and IV (Bolea-Alamañac et al., 2014). Guanfacine remains the preferential α2 adrenergic agonist with about 50–60% of children responding favorably as placed alongside relative efficacy with other non-stimulant drugs and effective in treating youths with symptoms of hyperactive/impulsive-inattentive (evidence level Ia) (Biederman et al., 2008; Bolea-Alamañac et al., 2014). In youths with ADHD, a medium effect size for efficacy and tolerability were reported, but these have to be weighed against possible adverse events of fatigue, somnolence/sedation, hypotension, bradycardia, and possible QTc prolongation (Hirota et al., 2014). Clarity of dose-response relationship for efficacy and tolerability outcomes are greatly needed using head-to-head controlled studies that compare α2 adrenergic agonists with stimulants and atomoxetine (Hirota et al., 2014).

##### Atomoxetine

Atomoxetine [ATM; IUPAC name: (3R)-*N*-methyl-3-(2-methylphenoxy)-3-phenylpropane-1-amine; brand name Strattera] is a noradrenergic antagonist drug that inhibits reuptake of NE by blocking NETs (Witcher et al., 2003; Corman et al., 2004), leading to an increase in NE in the synaptic cleft (Figure 3). In addition, ATM has a low affinity for serotonin and DA transporters that also inhibit reuptake of these monoamines across certain brain regions, such as the PFC (Bymaster et al., 2002; Sauer et al., 2005; Ding et al., 2014). For example, in animal studies, atomoxetine was shown to selectively increase dopamine in the PFC, while not altering areas rich with dopamine innervations such as the striatum (Bymaster et al., 2002; Swanson et al., 2006). Atomoxetine is the main non-stimulant drug that is recommended for treatment of ADHD in adults (Bolea-Alamañac et al., 2014). Clinical efficacy of stimulants and atomoxetine are comparable (Bolea-Alamañac et al., 2014; Shang et al., 2016) in adult ADHD (Durell et al., 2013; Fredriksen et al., 2013) and pediatric ADHD (Cheng et al., 2007), although they cannot be considered similar because they have differences in their mechanisms of action and hazards (Spencer et al., 1998; Bolea-Alamañac et al., 2014). Atomoxetine is preferred if there are any contra-indications to stimulant treatment, such as if methylphenidate is ineffective or not tolerated, presence of anxiety disorder or tics, and risk of misuse or diversion (Bolea-Alamañac et al., 2014). The presence of family history of cardiac problems or significant cardiovascular concerns should be monitored carefully (Bolea-Alamañac et al., 2014), such as cases with prolonged QT interval (Štuhec and Švab, 2013) and hypertension (Bolea-Alamañac et al., 2014). A systematic and expanded review has shown atomoxetine to be most effective in the treatment of ADHD symptoms in child, adolescent, and adults with comorbidity to anxiety and oppositional defiant disorder, while mixed or limited findings were found for individuals with ADHD and comorbid substance-use disorders, autism spectrum disorders, dyslexia or reading disorder, depression, bipolar disorder, and Tourette syndrome (Hutchison et al., 2016).

##### Bupropion

The antidepressant and smoking cessation agent Bupropion [brand names: Zyban, Wellbutrin, Elontril; IUPAC name: 2-(tert-butylamino)-1-(3-chlorophenyl)propan-1-one] is an off-label, non-FDA approved use for treating ADHD (Ng, 2017). Efficacy and tolerability for bupropion reaches a level Ia (Bolea-Alamañac et al., 2014), indicating small benefits for children (Stuhec et al., 2015; Ng, 2017) and adults (Verbeeck et al., 2017). Thus, more randomized controlled trials with larger sample sizes are warranted for a better clinical evaluation of bupropion. Bupropion is often used if methylphenidate, atomoxetine, and amphetamines have poor clinical response or if risk of stimulant abuse is high (Bolea-Alamañac et al., 2014). Contraindications include seizure disorder and suicidal ideation (Huecker et al., 2021). Compared to classical tricyclic antidepressants, bupropion mechanism of action serves as a norepinephrine-dopamine disinhibitor (Khan et al., 2016) and as a non-competitive antagonism of nicotinic acetylcholine receptors (Potter et al., 2006; Weiss et al., 2007; Verbeeck et al., 2017). Reuptake inhibition is high for dopamine, while norepinephrine uptake is less potent (Huecker et al., 2021).

## Neuroimaging alterations in attention-deficit/hyperactivity disorder: Medicated versus medication naïve

Structural and functional neuroimaging techniques permit non-invasive imaging of the anatomy and physiology of the human brain *in vivo*. In this section, we will review structural and functional brain differences in youths and adults who are ADHD medication naive, ADHD medicated with pharmacotherapy, and typically developing (TD) controls, as measured by (1) PET, (2) structural MRI, and (3) DTI at drug naivety and pharmacotherapy intervention.

### Positron emission tomography

#### Attention-deficit/hyperactivity disorder drug naïve

Radioactive tracers used in PET imaging often compete with endogenous ligands (e.g., dopamine), and measurements therefore can be used to determine the number of available binding sites for specific receptors (Muehllehner and Karp, 2006). Radiotracers commonly used to study ADHD include those that bind with dopaminergic presynaptic terminals (i.e., radiotracers: [^99M^Tc]TRODAT, [^11^C]PE2I, [^11^C]cocaine, [^11^C]-altropane), L-amino acid transporters (i.e., radiotracers: [^18^F]DOPA), and D_2_/D_3_ receptors (i.e., radiotracer [^11^C]raclopride) (Fusar-Poli et al., 2012). Compared to controls, there is increased radiotracer binding to striatal DATs in adults with ADHD (Dresel et al., 2000; Spencer et al., 2007), suggesting less DA in the synaptic cleft due to high levels of DA reuptake. Similarly, relative to controls, ADHD adult men had greater d-amphetamine-induced decrease in striatal [(11)C]raclopride binding, and the magnitude of this change was associated with poor response inhibition (Cherkasova et al., 2014). In addition, adults with ADHD have low D_2_/D_3_ receptor binding potential across the left hemisphere of the caudate, nucleus accumbens, midbrain, and hypothalamus (Volkow et al., 2007, 2009), suggesting low levels of D_2_/D_3_ receptors. Interestingly, low densities of D_2_/D_3_ receptors in the nucleus accumbens are also associated with greater risk for drug abuse (Dalley et al., 2007). In another PET study by Volkow et al. (2002), there was an attenuation of DAT and D3/D4 receptor availability in the nucleus accumbens and midbrain from adults with ADHD compared to individuals without ADHD, and this reduction was associated with low motivation. Adolescents with ADHD also have decreased DAT binding potential in the midbrain suggesting altered DA signaling, and DA 2 receptor binding in the right caudate correlated with greater motor hyperactivity (Jucaite et al., 2005).

#### Attention-deficit/hyperactivity disorder medicated

Positron emission tomography (PET) studies have generally found that stimulant treatment for ADHD tends to increase the rate of dopamine release into synapses from striatal brain regions. However, few studies have examined children due to concerns about PET radiation. A meta-analysis of 9 PET and 10 SPECT studies revealed that patients with ADHD had 14% greater striatal DAT density than in TD, but this was influenced by previous exposure to ADHD medication with lower DAT density in medication naïve ADHD subjects (Fusar-Poli et al., 2012). Methylphenidate (MPH) blocks DAT sites in the striatum of adults with ADHD compared to controls, leading to increased DA in the extracellular space (Dresel et al., 2000). Ludolph et al. (2008) found lower radiotracer binding for DATs in MPH-treated adults in the striatum, suggesting a down-regulation of DA turnover. In children, adolescents, and adults with ADHD, a reduction of binding potential is seen in striatal D_2_/D_3_ receptors after intake of MPH, which may reflect increased extracellular DA (Rosa-Neto et al., 2005; Volkow et al., 2007). The amount of extracellular DA regulated by psychostimulants depends on a combination of the DAT blockade and the rate of DA release, as well as individual differences in cell firing and stimulation (Volkow et al., 2005). For example, one PET study noted a possible physiological explanation for drug tolerance, where an increase in DATs was seen in adults with ADHD who had received less than 12 months of MPH treatment (Wang et al., 2013).

### Structural magnetic resonance imaging

#### Attention-deficit/hyperactivity disorder drug naïve

A range of structural brain alterations have been reported in structural magnetic resonance imaging (sMRI) studies examining children and adults with ADHD at baseline (medication naïve). These abnormalities are often reported in the frontostriatal circuitry (Emond et al., 2009; Cubillo et al., 2012; Norman et al., 2016), but also affected regions include fronto-parieto-temporal, fronto-cerebellar, and fronto-limbic networks (Seidman et al., 2005; Rubia et al., 2014). These multiple systems are implicated in attention, cognitive control, and working memory (Arnsten and Rubia, 2012). Meta-analyses of ROI or whole brain sMRI volumetric studies of ADHD consistently report overall reduction of total or right cerebral volumes (Valera et al., 2007; Nakao et al., 2011), right (Valera et al., 2007) or bilateral caudate (Nakao et al., 2011; Frodl and Skokauskas, 2012), right (Ellison-Wright et al., 2008; Norman et al., 2016) or bilateral putamen (Frodl and Skokauskas, 2012), right globus pallidus (Ellison-Wright et al., 2008; Frodl and Skokauskas, 2012), right lentiform nucleus (Nakao et al., 2011), posterior and inferior cerebellar vermis (Valera et al., 2007), splenium of the corpus callosum (Valera et al., 2007), anterior cingulate cortex (Seidman et al., 2006; Frodl and Skokauskas, 2012), whereas greater volume has been found in the left posterior cingulate cortex (Nakao et al., 2011). Other observed abnormalities in gray matter volume have been found in prefrontal and frontal areas, temporal, occipital, and parietal cortices (Mostofsky et al., 1998; Carmona et al., 2005; McAlonan et al., 2007; Valera et al., 2007; Ahrendts et al., 2011; Nakao et al., 2011; Castellanos and Proal, 2012; Lopez-Larson et al., 2012; Bralten et al., 2016; Norman et al., 2016; Gehricke et al., 2017; Lu et al., 2019; Wu et al., 2019), with some studies indicating greater volumes than controls in frontal (Semrud-Clikeman et al., 2017; Lu et al., 2019; Wu et al., 2019) and caudate regions (Semrud-Clikeman et al., 2017). In the largest cross-sectional study conducted to date on subcortical brain volumes of ADHD, Hoogman et al. (2017) found significantly smaller volumes for the accumbens, caudate, amygdala, hippocampus, and putamen bilaterally. This confirms other sMRI studies on morphology abnormalities of the amygdala (Plessen et al., 2006; Frodl et al., 2010; Douglas et al., 2018), hippocampus (Plessen et al., 2006; Douglas et al., 2018), caudate (Douglas et al., 2018), and thalamus (Douglas et al., 2018). Regardless of these significant findings, a recent meta-analysis using activation likelihood estimation reported no significant convergent structural MRI alterations in children and adolescents with ADHD, highlighting the need to explore homogenous clinical samples and analyses (Samea et al., 2019). In contrast, adults with ADHD are shown to have normal prefrontal, striatal, and parietal gray matter volumes (Ahrendts et al., 2011). Differences in ADHD presentation phenotypes have been found. The right inferior frontal gyrus in young adults (Depue et al., 2010) and cerebellar vermis lobes VIII–X (Bledsoe et al., 2009) in children with ADHD combined phenotype are found to be reduced in volume. Decreased morphology volumes in the right lateral and left posterior thalamic surfaces (associated with hyperactivity) and increased volumes in the right medial thalamic surfaces (associated with inattention) (Ivanov et al., 2010) are shown to differentiate ADHD behavioral profiles.

Similar to volumetric results, there were significant findings in cortical surface area and cortical thickness metrics. Lower surface area values were found in children with ADHD, localized to the frontal, cingulate, and temporal areas (Wolosin et al., 2009; Mous et al., 2014; Silk et al., 2016; Ambrosino et al., 2017; Hoogman et al., 2019), as well as less rightward asymmetry for total hemispheric and medial orbitalfrontal cortex surface area (Postema et al., 2021). However, studies on gyrification or intrinsic curvature of surface area showed inconsistent findings whereby controls and children/young adults with ADHD did not show significant differences (Shaw et al., 2012; Forde et al., 2017). This suggests that cortical abnormalities in development are related to differential brain expansion across subjects (Forde et al., 2017). Additionally, rather than thinning of the cortex, Ambrosino et al. (2017) suggested that cortical volume decreases were driven primarily by surface area reductions. On the other hand, decreases in cortical thickness (CT) were observed throughout the cortex (Narr et al., 2009) and the magnitude of CT decrease appears to correlate with disease severity (Almeida et al., 2010). These CT reductions are found across the cortex, particularly in frontal regions (Sowell et al., 2003; Makris et al., 2007; Almeida et al., 2010; Qiu et al., 2011; Shaw et al., 2011; Almeida Montes et al., 2013; Silk et al., 2016), temporal (Hoogman et al., 2019), parietal (Makris et al., 2007; Almeida Montes et al., 2013; Silk et al., 2016), and occipital (Hoogman et al., 2019) across children and adults with ADHD (Albajara Sáenz et al., 2019). In contrast, increased cortical thickness was also found in a group of children/adolescents with ADHD within the occipital lobe (Almeida Montes et al., 2013). These studies evidence the complexity of structural abnormalities in medication naïve ADHD subjects across all age groups.

#### Attention-deficit/hyperactivity disorder neurodevelopment during drug naivety

Anatomical MRI studies of ADHD are often cross-sectional, but prospective longitudinal studies have enabled researchers to detect patterns of aberrant developmental trajectories in ADHD groups compared to healthy subjects. It has long been argued that ADHD children have a delay in brain maturation (Shaw et al., 2007, 2012) due to late developing fronto-striatal and fronto-cerebellar systems (Rubia, 2013) that diminish ADHD symptoms in later adulthood. Shaw et al. (2007, 2012) found delays in peak cortical thickness and surface area development by 2–5 years in children with ADHD, with the most prominent delay in the prefrontal region that controls cognitive processes of motor and attention planning. This pattern of persistent reductions in frontal cortices of volume, surface area, and gyrification among ADHD subjects aged 6–28 were replicated in a recent study (Ambrosino et al., 2017). Age-related changes in the frontal eye field (L-FEF) and left ventral frontal cortex (L-VFC) were detected in children with ADHD (Lu et al., 2019). A decrease in surface area of the L-VFC and an increase in volume of the L-FEF persists in children with ADHD (7–16 years) (Lu et al., 2019). Besides frontal cortices, growth is also seen to be stunted in other regions. Age related growth in gray matter of bilateral occipital lobe appears reversed in children with ADHD (Wu et al., 2019). Stable symptoms of ADHD over an average of 4.8 years in a cohort of 362 youths was associated with reduction of thalamic volume (Sudre et al., 2021). Progressive atypical contraction was found in ventral and dorsal striatal regions that persisted into adolescence for the ADHD group compared to surface area expansion with age in the typically developing group (Shaw et al., 2014). On the other hand, not all studies ascertain these findings. Volumetric abnormalities in ADHD have been shown to normalize or decrease in childhood (Wu et al., 2019), adolescence (Castellanos et al., 2002), and adulthood (Perlov et al., 2008; Greven et al., 2015; Hoogman et al., 2017) compared to controls in the hippocampus (Perlov et al., 2008; Hoogman et al., 2017), amygdala (Perlov et al., 2008; Hoogman et al., 2017), accumbens (Hoogman et al., 2017), putamen (Greven et al., 2015; Hoogman et al., 2017), caudate (Castellanos et al., 2002; Greven et al., 2015; Hoogman et al., 2017), and medial frontal regions (Wu et al., 2019).

Nevertheless, persistent brain abnormalities are found in adult ADHD and are elevated due to severity levels, behavioral profiles, environmental factors. Adults with childhood ADHD showed sustained dysfunctions in the lateral fronto-striatal and ventromedial orbitofrontal during attention- and reward-related tasks similar to pediatric ADHD (Cubillo et al., 2012). Rate of cortical thinning in the medial and dorsolateral PFC has been associated with persistent inattentive rather than hyperactive/impulsive symptoms in adult ADHD, whereas cortical thickening or minimal thinning was found among ADHD adult subjects who remitted (Shaw et al., 2013). Interestingly, inattentive symptoms in healthy children have previously been associated with decreased regional thickness and thinning rate in the right lateral and left medial PFC (Ducharme et al., 2012). These results are in consonant with another study done by Shaw et al. (2011) where typically developing children with high levels of hyperactivity/impulsivity showed slow rate of cortical thinning among prefrontal, premotor, and cingulate regions. Additionally, ADHD subjects show a non-progressive loss of volume in the superior cerebellar vermis from childhood to adolescent years regardless of clinical outcome (Mackie et al., 2007). However, a downward trajectory in volumes of bilateral inferior-posterior cerebellar lobes are exhibited in ADHD subjects who have worst clinical outcomes (Mackie et al., 2007; Leech and Sharp, 2014). Evidence of severe early-life deprivation (by institutionalization) from a cohort of children with ADHD results in reduced cortical thickness across the lateral orbitofrontal cortex, insula, inferior parietal cortex, precuneus, superior temporal cortex, lingual gyrus, supramarginal gyrus, and fusiform gyrus (McLaughlin et al., 2014) compared to age-matched community control subjects. These regions were also found to be associated with inattention and impulsivity (McLaughlin et al., 2014). Future neurodevelopmental studies of anatomy may shed light on the clinical presentations of ADHD by considering longitudinal cohort designs that account for phenotypic heterogeneity. Besides these factors that modulate the structural abnormalities in the ADHD brain, exposure to stimulant medication also alternates these brain signatures.

#### Attention-deficit/hyperactivity disorder medicated

There is mounting evidence to suggest neural anatomical alterations in ADHD as a result of psychotherapeutic intervention (Spencer et al., 2013; Chou et al., 2015). Numerous cross-sectional studies have examined the effects of psychostimulant treatment on ADHD. We first discuss cross-sectional studies that quantified volumetric alterations in the ADHD brain over the course of pharmacotherapy, followed by a discussion of morphometry results. Although there are relatively fewer longitudinal studies, we summarize extant literature of studies examining pre/post medications effects on structural MRI measures.

##### Volumetric cross-sectional studies

Overall voxel-based morphometry (VBM) studies have yielded mixed results when considering the effects of stimulant medication on brain structure. In general, studies that reported significant effects suggest that certain subcortical and cortical nuclei are normalized in medicated ADHD children compared to medication naïve ADHD children. A meta-regression analysis found no association between gray matter volume abnormalities and long-term stimulant medication use from ADHD patients across childhood and adulthood ages (Norman et al., 2016). Villemonteix et al. (2015) reported a volume reduction in middle frontal and precentral gyrus in treatment naïve ADHD children compared to both medicated children with ADHD and controls (Villemonteix et al., 2015). For subcortical areas, Semrud-Clikeman et al. (2006) found an association between duration of treatment and normalized GM volume in the caudate and left nucleus accumbens (ACC) in children with ADHD. Similarly, the right anterior cingulate cortex was found to be normalized in children and adolescents with a treatment history in ADHD-combined (Semrud-Clikeman et al., 2014).

Contrary to results that showed brain normalization, Schweren et al. (2015) found that a combined treatment group (methylphenidate and antipsychotics) of adolescents with ADHD results in reduction of total cortical volume, bilateral ventral diencephalon, and left thalamus compared to healthy controls. This was not found in the group that were only medicated with methylphenidate (Schweren et al., 2015). Thus, the findings may indicate that antipsychotic treatment could counteract the normalizing effects of methylphenidate on brain structure, but the authors are quick to note that they did not have an untreated ADHD group which hinders interpretation of the results (Schweren et al., 2015). Furthermore, another cross-sectional study found that current stimulant use versus no current use was associated with lower surface area in two frontal cortex regions (Hoogman et al., 2019).

Interhemispheric laterality findings of stimulant medication have been mixed. Patterns of absolute asymmetry volumes appear to increase in the caudal anterior cingulate and isthmus cingulate for medicated ADHD youths compared to medication naïve ADHD youths (Douglas et al., 2018). In a longitudinal replication design, increase absolute asymmetry volume was greater in medication naïve than medicated ADHD youths in cortical regions of the frontal, occipital, parietal, and temporal (Dutta, 2020). Postema et al. (2021) found current medication use was associated with surface area asymmetries in the precuneus and transverse temporal, and with thickness asymmetries in the inferior parietal cortex and precentral. Lifetime psychostimulant medication use involved asymmetries of surface area insula, supramarginal gyrus, and rostral anterior cingulate cortex, and thickness asymmetry of the paracentral lobule (Postema et al., 2021). The asymmetry of precentral gyrus thickness was associated with an ADHD diagnosis across all age groups (Postema et al., 2021). Thus, it appears that stimulants both increase and decrease structural brain asymmetries.

In contrast, other cross-sectional studies have found no volumetric differences in the ADHD brain as a result of medication intake. Greater (Semrud-Clikeman et al., 2017) or smaller (Castellanos et al., 2002; Semrud-Clikeman et al., 2006) volumes of the caudate were no different based on medication history/status in children and adolescents with ADHD. These also translated to other regional volumetric findings in the cerebellum (Castellanos et al., 2002), right prefrontal (Semrud-Clikeman et al., 2017), bilateral anterior cingulate cortex (Semrud-Clikeman et al., 2006), insula and middle temporal gyrus (Villemonteix et al., 2015). A cross-sectional mega-analysis on subcortical nuclei found that stimulant medication for ADHD was not related to structural changes across the lifespan, noting that the effects may be too local to be picked up by volumetric analysis (Hoogman et al., 2017). Indeed, morphology analyses may provide more insight into regional differences.

##### Morphology studies

A variety of studies suggest that more localized morphological alterations appear to resolve or normalize after treatment with stimulants that enhance DA signaling. For example, Sobel et al. (2010) found attenuation of morphology deformations in specific basal ganglia regions in ADHD medicated children, suggesting that stimulants may normalize morphological differences in the caudate, putamen, and globus pallidus. Sub-regions of the cerebellum, such as the posterior inferior vermis, show reduced attenuation in ADHD medicated children compared to medication naïve subjects (Berquin et al., 1998; Bledsoe et al., 2009). Morphology analyses from youths (children and adolescents) with ADHD reveal increased volumes in the anterior cingulate cortex (ACC) (Semrud-Clikeman et al., 2006), anterior thalamic pulvinar (Ivanov et al., 2010), splenium of the corpus callosum (Schnoebelen et al., 2010), and left lateral cerebellar surface (Ivanov et al., 2014) associated with intake of stimulant treatment. On the other hand, follow-up analyses revealed atypical surface area morphology in the PFC for ADHD children prescribed with stimulant medication (Dirlikov et al., 2015).

##### Longitudinal studies

**FIGURE 4:**
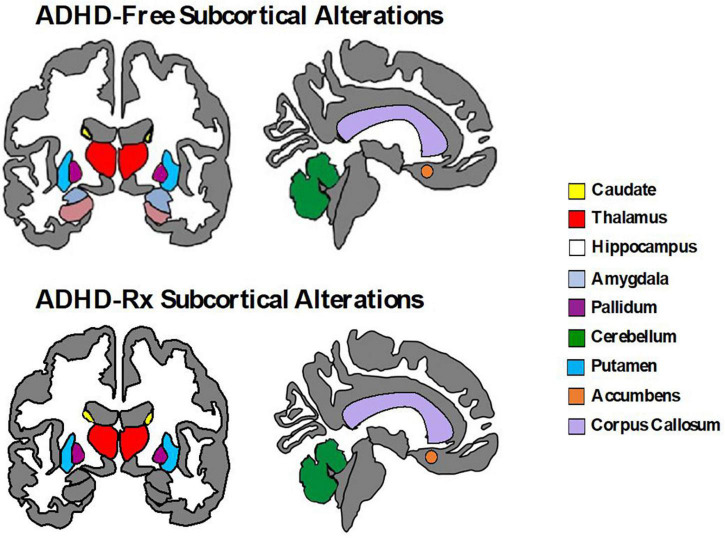
Subcortical brain alterations in ADHD-free (medication naïve) and ADHD-Rx (medicated). These figures were created using *ggseg* library from R program (Mowinckel and Vidal-Piñeiro, 2020).

**FIGURE 5:**
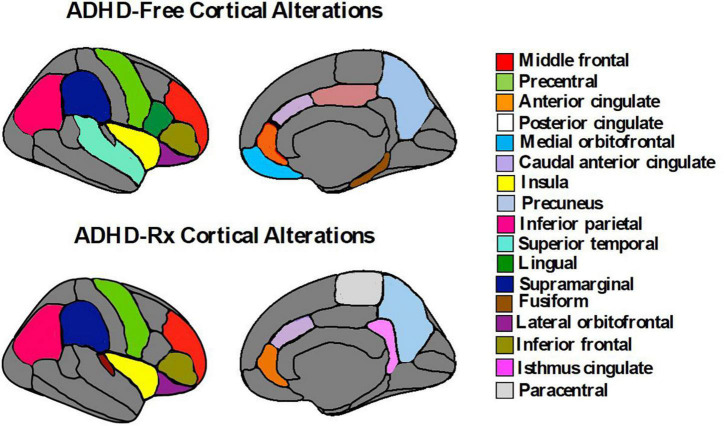
Cortical brain alterations in ADHD-free (medication naïve) and ADHD-Rx (medicated). These figures were created using *ggseg* library from R program (Mowinckel and Vidal-Piñeiro, 2020).

Relatively fewer studies have investigated longitudinal changes before and after medication onset prospectively. Some evidence suggests that the age of medication onset and treatment duration may play a role in predicting the magnitude of medication effects on brain structure and function. For example, Shaw et al. (2009b) found the rate of change between adolescents (12.5–16.4 years) with ADHD taking psychostimulants differed from those not taking psychostimulants in cortical thickness measures of the right motor strip, left middle/inferior frontal gyrus, and right parieto-occipital region, but importantly, this was not associated with clinical outcome. They found cortical thinning was more pronounced in the group not taking psychotherapy (0.16 mm/year) compared to the group taking psychotherapy (0.03 mm/year) (Shaw et al., 2009b). On the contrary, no association between psychostimulant medication and the development of the surface morphology for the basal ganglia (caudate, putamen, globus pallidus) was found (Shaw et al., 2014). Instead, the ADHD group (childhood to adolescence), regardless of medication status, showed progressive contraction of the ventral striatal surfaces (1.75 mm^2^ per year) compared to controls (rate of increase 0.54 mm^2^ per year) (Shaw et al., 2014). In adults with ADHD, before and after 3 years of psychostimulant treatment, left putamen GM volumes are similar to controls, but increased compared to non-medicated ADHD adults suggesting a normalizing effect (Pretus et al., 2017). Similarly, after 1–2 years of MPH treatment, adult ADHD patients revealed recovered nucleus accumbens (Nacc) gray matter volumes relative to controls (Hoekzema et al., 2014). van Elst et al. (2016) observed an increase in cerebellar GM volume for MPH-treated adults with ADHD after a year of treatment, but no change in cortical thinning. Two meta-regression analyses of VBM studies (Nakao et al., 2011; Frodl and Skokauskas, 2012) and one qualitative review (Spencer et al., 2013) examined long-term psychostimulant effects. These studies found that long-term stimulant medication use was associated with normalized basal ganglia volumes (Nakao et al., 2011; Frodl and Skokauskas, 2012; Spencer et al., 2013) thus suggesting no evidence that stimulant drugs cause abnormal brain development in ADHD (Spencer et al., 2013). Another longitudinal study found brain volume asymmetry decreases across the cortex (frontal, temporal, occipital, and parietal) with stimulant medication (8 weeks exposure to MPH and/or guanfacine) compared to medication naïve ADHD youths (Dutta, 2020). Thus, it is likely that the impact of psychostimulants on neurodevelopment is enabling anatomic normalization (Friedman and Rapoport, 2015). Figure 4 displays subcortical alterations seen in ADHD medication naïve subjects and ADHD-Rx (medicated), and Figure 5 images cortical changes seen in medication naïve and treated ADHD subjects.

### Diffusion tensor imaging

#### Attention-deficit/hyperactivity disorder drug naïve

**FIGURE 6:**
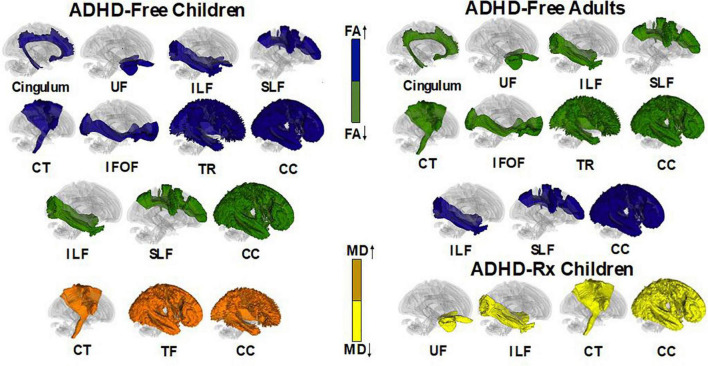
Illustrative figure of tractography fibers showing significant differences across ADHD treatment naïve and treated individuals. In ADHD-Free (treatment naive), subjects had differences of fractional anisotropy (FA) and mean diffusivity (MD) in the following white matter regions: cingulum (decrease FA in adults and increase FA in children; Makris et al., 2008; Konrad et al., 2010; Svatkova et al., 2016), uncinate fasciculus (UF) (increase FA in children and decrease FA in adults; Silk et al., 2009b; Shaw et al., 2015), inferior longitudinal fasciculus (ILF) (increase and decrease of FA in children/adults), superior longitudinal fasciculus (SLF) (increase and decrease in FA in children/adults; Hamilton et al., 2008; Makris et al., 2008; Pavuluri et al., 2009; Silk et al., 2009b; Kobel et al., 2010; Liston et al., 2011; Nagel et al., 2011; van Ewijk et al., 2012; Cortese et al., 2013; Lawrence et al., 2013; Svatkova et al., 2016), corticospinal tract (CT) [decrease FA in adults and increase FA in children; increase MD in children; Hamilton et al., 2008; Luis-García et al., 2015; Svatkova et al., 2016; included are white matter regions of the *superior, anterior, and posterior corona radiata* (decrease FA for children/adolescents/adults; Pavuluri et al., 2009; Kobel et al., 2010; Nagel et al., 2011; Qiu et al., 2011; Cortese et al., 2013), *the anterior and superior limb of the internal capsule* (decrease FA in children/adolescents/adults; Pavuluri et al., 2009; Silk et al., 2009b; Nagel et al., 2011; Qiu et al., 2011; van Ewijk et al., 2012; Cortese et al., 2013), and *the cerebellar peduncle* (decrease/increase FA in children; increase FA in adolescents; Ashtari et al., 2005; Makris et al., 2008; Bechtel et al., 2009; Kobel et al., 2010; Nagel et al., 2011)], inferior fronto-occipital fasciculus (IFOF) [included is the sagittal stratum (decrease FA in adults; increase FA in children; Peterson et al., 2011; Cortese et al., 2013)], anterior and superior thalamic radiation (decrease FA in adults; increase FA in children/adolescents; increase MD in children/adolescents; Silk et al., 2009b; Cortese et al., 2013; Svatkova et al., 2016), and corpus callosum [included white matter tracts include *the splenium, genu, isthmus* (decrease FA in children/adolescents/adults; increase FA in children/adolescents; increase MD in children/adolescents; Chao et al., 2009; Pavuluri et al., 2009; Cao et al., 2010; Peterson et al., 2011; Qiu et al., 2011; Dramsdahl et al., 2012; Luis-García et al., 2015)] and *the forceps minors* (decrease FA in children; Qiu et al., 2011; van Ewijk et al., 2012; Lawrence et al., 2013; Svatkova et al., 2016). ADHD-Rx (treated) children had decreases in MD for the following white matter tracts (Luis-García et al., 2015): unicinate fasciculus, inferior longitudinal fasciculus, corticospinal tract, and corpus callosum (sector IV). All images were created using DTI data from DTI Studio (Jiang et al., 2006; Fedorov et al., 2012).

Diffusion tensor imaging (DTI) examines direction and displacement of water molecules in the brain in order to infer white matter (WM) architecture (Basser et al., 1994). Region of interest (ROI) studies have found asymmetry in WM integrity in ADHD individuals. Figure 6 displays each of these regions along with their asymmetries in fractional anisotropy and mean diffusivity found in ADHD children and adults: the superior and inferior longitudinal fasciculus (Hamilton et al., 2008; Makris et al., 2008; Pavuluri et al., 2009; Silk et al., 2009b; Kobel et al., 2010; Liston et al., 2011; Nagel et al., 2011; van Ewijk et al., 2012; Cortese et al., 2013; Lawrence et al., 2013; Svatkova et al., 2016); the anterior, posterior, and superior of the corona radiata (Pavuluri et al., 2009; Kobel et al., 2010; Nagel et al., 2011; Qiu et al., 2011; Cortese et al., 2013); posterior and anterior thalamic radiation (Silk et al., 2009b; Cortese et al., 2013; Svatkova et al., 2016); cerebellum and cellebellar peduncle (Ashtari et al., 2005; Makris et al., 2008; Bechtel et al., 2009; Kobel et al., 2010; Nagel et al., 2011); the splenium, isthmus, and genu of the corpus callosum (Chao et al., 2009; Pavuluri et al., 2009; Cao et al., 2010; Peterson et al., 2011; Qiu et al., 2011; Dramsdahl et al., 2012) and the posterior and anterior limb of the internal capsule (Pavuluri et al., 2009; Silk et al., 2009b; Nagel et al., 2011; Qiu et al., 2011; van Ewijk et al., 2012; Cortese et al., 2013). Voxel-based analyses (VBA) also confirm ADHD white matter deficits in: the unicinate fasciculus (Silk et al., 2009b; Shaw et al., 2015); forceps minor (Qiu et al., 2011; van Ewijk et al., 2012; Lawrence et al., 2013; Svatkova et al., 2016); corticospinal tract (Hamilton et al., 2008; Svatkova et al., 2016); cingulum bundle (Makris et al., 2008; Konrad et al., 2010; Svatkova et al., 2016) and sagittal stratum (Peterson et al., 2011; Cortese et al., 2013). Abnormalities may depend on ADHD presentation, where inattention has been linked to impairments in frontostriatal circuits while hyperactivity has been linked to impairments in frontolimbic circuits (Konrad and Eickhoff, 2010; Lei et al., 2014; Svatkova et al., 2016). These results parallel the dual pathway model of ADHD, suggesting that frontostriatal pathways lead to executive dysfunction while frontolimbic pathways lead to rewarding response and motivation deficits (Sonuga-Barke, 2003, 2005; Castellanos et al., 2006).

#### Attention-deficit/hyperactivity disorder medicated

The impact of ADHD medication on WM microstructure remains unclear. Ashtari et al. (2005) found no significant effects on FA values between ADHD-Rx (medicated) and ADHD-Free (medication naïve) groups in six brain regions. Luis-García et al. (2015) found MD was reduced in methylphenidate (MPH) medicated ADHD patients in fronto-striatal WM tracts (Luis-García et al., 2015). In their longitudinal study, de Zeeuw et al. (2012) found a decrease of FA for all ADHD groups regardless of MPH treatment duration. Asymmetry patterns for FA measures of the uncinate fasciculus and inferior lateral fasciculus appear to normalize with stimulant medication compared to ADHD-Free (medication naïve) subjects (Douglas et al., 2018). Figure 6 displays fiber regions that were found to differ in ADHD children and adults when under psychostimulant treatment and medication naive.

## Neurocomputational theories of catecholamines and attention

Catecholaminergic brain systems, such as dopamine (DA) and norepinephrine (NE), are important neuromodulators that control attention. In light of their shared biosynthesis, intracellular signaling, and innervation pathways, it is critically important to differentiate their functions within a unified paradigm to explain the pathophysiology of ADHD. Computational psychiatry attempts to develop neurocomputational models that can describe the cognitive deficits typical of ADHD as they relate to brain function. While extent attempts are scarce, they are promising (see Williams and Dayan, 2005; Frank et al., 2007a,b; Luman et al., 2010; Hauser et al., 2016; Ziegler et al., 2016). Behavioral profiles of ADHD have been differentiated given current neurocomputational models: A hyperactive/impulsivity presentation can be expressed as a behavioral switching between less valuable options, whereas an inattentive presentation is a shift between goal orientation and an inability to stay focus on one individual goal (Hauser et al., 2016). The catecholaminergic profiles of each ADHD presentation is still difficult to disentangle though the clear distinction is made between dopamine and norepinephrine action selection models that could explain the overarching diagnosis. A decrease in ‘dopamine’ precision leads to high entropy or to many surprising events for the brain to process, whereas ‘norepinephrine’ heightens learning to both typical and novel information thus increasing attentional variability. Here, we merge computational, algorithmic, and implementation Marrian levels of analysis (Marr, 2010) to demonstrate how catecholamines can be dissociated to explain ADHD phenotype.

### Dopamine

Dopamine was discovered as a neurotransmitter in 1957 by neuropharmacologist Arvid Carlsson during exploits of DA in the basal ganglia and motor function in patients with Parkinson’s disease (Carlsson, 1959, 1993). Interest in dopamine as a mechanism for reward-based learning and motivation gained momentum through use of electrophysiological recordings and pharmacological manipulations of DA in animals (Olds and Milner, 1954; Wise, 1982; Schultz, 1986; Schultz et al., 1993; Viggiano et al., 2003, 2004). These early experiments uncovered two patterns of DA firing: tonic and phasic activity (Schultz, 1986, 2001). Tonic firing patterns consist of slow and sustained extracellular DA neuron firing, while phasic activation consists of sudden firing rate change (50–110 ms; duration < 200 ms) of DA concentrations. Intermediary levels of firing reflect slow burst firing lasting seconds to minutes (Schultz, 2001). Current theoretical neurocomputational models examine phasic and tonic DA signaling in select brain regions. One such model, reinforcement learning theory (Sutton and Barto, 1998), proposes an adaptive decision-making framework based on optimizing behavior to obtain future reward or avoid punishments. This trial-and-error learning process is established under four main schemes (Sutton and Barto, 1998; Montague et al., 2004; Dayan and Daw, 2008; Samson et al., 2010; Daw and Tobler, 2014): (1) a reward function or value to a state, (2) a weighted running average update that accounts for all rewards received previously in the presence of the stimulus with the most recent reward weighted heavily than the prior rewards, (3) a value function updating the prediction based on the current reward/state and direction of the weight, and (4) reward prediction error (RPE) comparing what reward the subject experiences on a specific trial and what reward they expected based on previous learning. The RPE reflects the rate of firing of D1 and D2 neurons in the ventral tegmental area (VTA) and the substancia nigra pars compacta (Schultz et al., 1997; Schultz, 2001; Lammel et al., 2012), striatum (Hernández-López et al., 1997, 2000; Daw et al., 2011; Watabe-Uchida and Uchida, 2018), lateral orbitofrontal cortex (Tobler et al., 2007), hippocampus and PFC (Gurden et al., 2000). Fast latency (50–110 ms) and duration (<200 ms) of phasic DA RPEs are observed during food/liquid rewards (animal experiments), conditioned reward-predicting stimuli (classical, simple choice RT, delayed go/no-go, visual discrimination tasks), and non-noxious stimuli that induce avoidance (Schultz, 2001). Dopamine depression or activation-depression responses are seen following stimuli that resemble the reward, following novel or intense stimuli, or during reward omission errors (Schultz, 2001).

Under some other accounts, dopamine neurons are postulated to signal ‘prediction error’ rather than reward. In this view, the brain makes inferences about the environment and tests these against sensory evidence in order to reduce free energy (i.e., prevent entropy or time average of surprise) (Friston, 2010; Friston et al., 2015, 2017; Parr and Friston, 2017). A Markovian decision process of a probabilistic generative model is considered: *P*(π) = σ(−γ⋅*G*(π)), whereby P prior distributions over policies π is equal to policies selected based on free energy G multiplied by ***an inverse temperature parameter*** −γ corresponding to precision of beliefs (i.e., dopamine firing) about policies over a softmax normalized exponential σ. Here, γ reflects the rate of DA firing. Encoding of dopamine as a ‘precision signal’ is illustrated in a number of empirical studies such as repetition suppression during learning (Bromberg-Martin and Hikosaka, 2009), hippocampal place cell activity during spatial tasks (Moser et al., 2015; Retailleau and Morris, 2018), and errors of omission and commission during oddball tasks (Bendixen et al., 2012). Besides its modulations in reward and precision, dopamine is ascribed to a number of other behaviors including belief and latent states (Rao, 2010), critic (ventral striatum) versus actor roles (dorsal striatum) (O’Doherty et al., 2004), hierarchical levels of abstraction learning when confronted with novelty (O’Reilly and Frank, 2006; Badre et al., 2010), judgment of time (Kurth-Nelson and Redish, 2009; Soares et al., 2016; Hamid et al., 2019), quality versus quantity stimuli attributes (de Berker et al., 2019), values for each effector (Gershman et al., 2009), and future events besides reward (Gardner et al., 2018).

**FIGURE 7:**
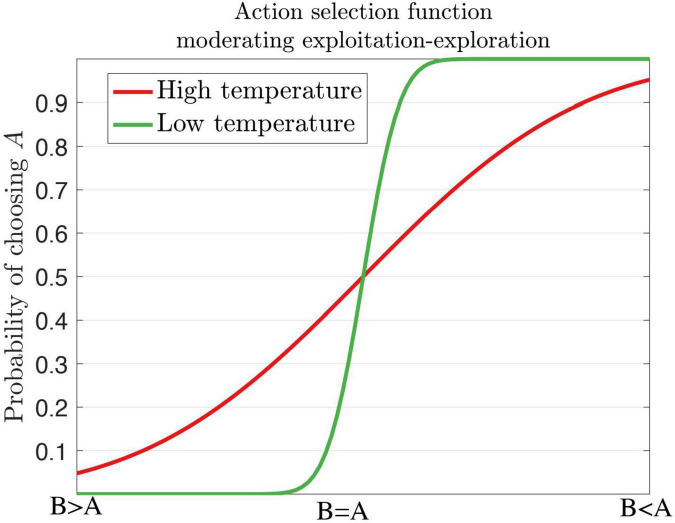
A softmax decision steepness parameter influences high and low temperatures for the action selection of dopamine. A high temperature renders variable behaviors as seen in ADHD, whereas a low temperature renders stable behaviors. Inspired by Hauser et al., 2016, Figure 1.

Several behavioral markers of ADHD can be explained by theories of reinforcement learning and precision error. For example, the decision temperature parameter determines if a subject will choose the optimal response or a variable response amongst all other alternative options (Herrnstein, 1961). Increasing the levels of the temperature parameter elicits variable and exploratory behavior, which is the case for ADHD. Individuals with ADHD do not always choose or exploit the best option, rather they exhibit response variability and response inconsistency (Hauser et al., 2016). Patients with ADHD display increased reaction time (RT) variability (Castellanos and Tannock, 2002; Tamm et al., 2012; Kofler et al., 2013) in working memory, and go/no-go and stop tasks with increased activity in the frontal regions and default mode network (DMN) (Uddin et al., 2008; Fassbender et al., 2009; Mohan et al., 2016). During the continuous performance task (CPT), ADHD subjects respond more often to non-target related responses (errors of omission) than target-related responses (errors of commission) (Losier et al., 1996; Huang-Pollock et al., 2012) with implicated circuits of the PFC, insula, and parietal areas. In the context of decision-making, individuals diagnosed with ADHD are more likely to choose immediate and small rewards across delay of gratification tasks, delay discounting tasks (Patros et al., 2016) and probabilistic reversal learning task in the medial PFC (Hauser et al., 2014). This could suggest poor reward prediction error signals (Tripp and Wickens, 2008). On the other hand, ADHD subjects are suggested to have divergent learning patterns by choosing suboptimal choices through exploratory behaviors as indicated by an increased decision temperature (Friston et al., 2014; Schwartenbeck et al., 2015b). We illustrate low and high temperature parameters in the context of exploitation-exploration action selection for dopamine (Figure 7).

### Norepinephrine/noradrenaline

Norepinephrine, or noradrenaline, has also been viewed from a computational neuroscience perspective. Similar to dopamine, tonic and phasic levels of firing are examined in the context of behavioral tasks. Within theory on precision (Yu and Dayan, 2005; Dayan and Yu, 2006; Parr and Friston, 2017), noradrenergic transmission is proposed as a signal of ‘unexpected uncertainty’ or volatility of state transitions. This theory stems from noradrenergic cell activity during task demands that induce unexpected changes, including novelty, the introduction of novel reinforcement agents, and reversing or eliminating prior contingencies (Bouret and Sara, 2005; Nieuwenhuis et al., 2005; Bouret and Richmond, 2015). Drug treatment using idazoxan, an a2 receptor antagonist that activates noradrenergic system, was effective only when rats had to change from spatial to visual cues during a maze navigation rather than during these tasks, suggesting NA’s role in attentional shift (Curet et al., 1987; Devauges and Sara, 1990). In monkeys, non-target stimuli that resulted in false alarm responses elicited phasic locus coeruleus (LC) activation during a vigilance discriminative task (Rajkowski et al., 2004). Pupillometry measures have been linked to noradrenergic activity (for a review, see Koss, 1986; Larsen and Waters, 2018) of the LC, brainstem (e.g., areas of colliculi), and cingulate cortex as seen in extracellular recordings in rhesus macaques (Joshi et al., 2016), and human fMRI BOLD activity during rest and the oddball paradigm (Bush et al., 1999; Bush, 2011; Murphy et al., 2014). Other important roles denoted by noradrenergic activity include arousal modulations (Berridge and Waterhouse, 2003; Berridge, 2008; Pattij et al., 2012; de Gee et al., 2017), increased reaction time variability coinciding with tonic levels of NA firing, and ‘network reset’ behavioral planning through phasic NA levels (Aston-Jones and Cohen, 2005; Bouret and Sara, 2005; Dayan and Yu, 2006; Llorente et al., 2006; Frank et al., 2007a; de Gee et al., 2017), and exogenous attentional set shifting by phasic (exploitation) and tonic (exploration) NA levels (Aston-Jones and Cohen, 2005; Gabay et al., 2011; Pattij et al., 2012; Mathot et al., 2014; Tervo et al., 2014). A recent computational approach to norepinephrine has been linked to learning rates (Sales et al., 2019) under the active inference model (Schwartenbeck et al., 2015a; Friston et al., 2017). In brief, they explore noradrenergic activity as a state-action prediction error that can evoke both ‘explore-exploit’ and ‘network reset’ behavioral modifications. Phasic LC responses are linked to prediction of a reward in a Go/No-Go paradigm with reversal contingencies, and high tonic levels of LC activity were elicited during exploration and task disengagement (Sales et al., 2019). Prediction errors are enabled through LC innervations, while belief updates or responses to state-action prediction errors are broadcast *via* ascending projections from LC to the cortex (e.g., frontal cortex, anterior cingulate cortex, dorsal PFC) (Gläscher et al., 2010; Karlsson et al., 2012; Tervo et al., 2014; Ebitz and Platt, 2015; Sales et al., 2019).

**FIGURE 8:**
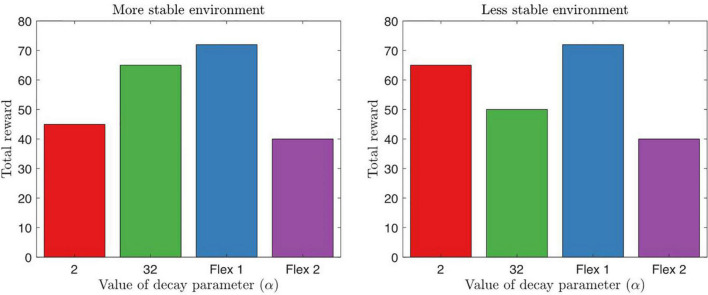
Here we illustrate a simple explore/exploit task for behavioral output with fixed alpha (α) learning parameters. α=32 represents a slow model decay learning with hyperflexible behavior to change strategies after one single failed trial, whereas α=2 represents a fast model decay where behavior is inflexible and persistence in behavior is shown. A less stable environment reflects changes in reward for every 10–15 trials, whereas a more stable environment reflects changes after 50–60 trials. We also display *flexible* alpha parameter that is able to adapt, however, flexible 1 is a behavioral output that enables an agent to rapidly switch in task demands, whereas flexible 2 displays an agent that varies behavior, but is not able to attain learning over time. A possible model of ADHD can reflect a variation of these learning parameters. Inspired by Sales et al., 2019, Figure 7.

Noradrenergic signaling influences attention through learning, saliency, and exploitative-exploratory behavioral avenues. NA action selection models, such as the decision temperature (Jepma and Nieuwenhuis, 2011; Eldar et al., 2016; Hauser et al., 2016) and learning rates (Sales et al., 2019), can describe ADHD phenotypes. Similar to dopamine, noradrenaline does affect response variability in ADHD, whereby high tonic but low phasic signaling is associated with reaction time variability (Aston-Jones and Cohen, 2005; Manev and Uz, 2009). The high tonic NA levels increase the representation of other stimuli cues in the environment, thus dissociating less strongly to high-valued options and preference for less optimal options (Servan-Schreiber et al., 1990; Eldar et al., 2013; Hauser et al., 2016). Evidence of NA dysfunction is reported in the prefrontal cortical region in rodent models of ADHD (Fan et al., 2011) and in human participants during response inhibition tasks (de Campo et al., 2011). The difference between noradrenaline learning and dopamine learning is that NA is associated with focus of relevant information and rate of learning, whereas dopamine is associated with precision of value that is ascribed to options. In an explore/exploit task, we illustrate total reward intake based on norepinephrine alpha parameters with slow, fast, and flexible model decays (Figure 8).

## Discussion

Pharmaceutical treatment for ADHD often leads to regional, but not global, brain alterations as observed across numerous structural neuroimaging modalities (Castellanos et al., 2002, 2008; Nakao et al., 2011; van Ewijk et al., 2012; Svatkova et al., 2016; Hoogman et al., 2017). Stimulants also seem to attenuate brain volume decreases and regional morphology asymmetries in basal ganglia across children, adolescents, and adults with ADHD (Semrud-Clikeman et al., 2006; Bledsoe et al., 2009; Shaw et al., 2009a; Ivanov et al., 2010; Schnoebelen et al., 2010; Sobel et al., 2010; Nakao et al., 2011; Villemonteix et al., 2015). Less is known about ADHD treatment effects on white matter structure since few systematic DTI treatment studies have been conducted to date (Ashtari et al., 2005; de Zeeuw et al., 2012; Luis-García et al., 2015). Smaller white matter volumes and asymmetric patterns in white matter microstructure are seen in both medicated and non-medicated ADHD youths (Castellanos et al., 2002; Douglas et al., 2018; Dutta, 2020), although these effects are more pronounced in non-medicated ADHD youths. It is possible this asymmetry may subtend the behavioral features of ADHD; however, such asymmetries may also serve as advantageous later in life.

In longitudinal PET and SPECT studies (van Dyck et al., 2002), typically developing individuals experience a 6–8% decline in striatal DATs per decade. Longitudinal studies in PET have not been conducted in ADHD populations but, typically, there is low DAT availability in non-medicated patients, whereas there is a high DAT availability in medicated patients among striatal regions. It is possible that either of these extremes can provide benefits toward striatal dopamine transporter integrity later in life. In essence, drug treatment for ADHD does not always appear to increase global brain volume or attenuate morphology abnormalities across all white and gray matter. Conversely, stimulant drugs are not suggested to cause abnormal development in ADHD populations.

Interestingly, with increasing age, certain brain regions tend to normalize on their own without the help of psychostimulant treatment. For example, caudate volume seems to normalize by mid-adolescence to early-adulthood in ADHD participants, both stimulant-treated and non-treated populations (Castellanos et al., 1994, 2002). In addition, some studies report comparable brain volumes between treatment-naïve and treated ADHD populations. As mentioned earlier, pharmacotherapy has frequently been seen to normalize brain regions within the basal ganglia (Sobel et al., 2010). However, with or without treatment, children and adolescence with ADHD have relatively smaller global gray matter brain volumes compared to healthy age-matched participants. The growth of these brain volumes, although smaller, parallel age-matched controls, reflecting fixed neurobiological abnormalities (Castellanos et al., 2002; Nakao et al., 2011). In late adulthood such brain volumes tend to remain constant regardless of treatment and comorbidity, whereas aged-matched controls experience a decrease in brain volume.

Despite the importance of these findings, most ADHD studies are confounded by relatively small sample sizes, cross-sectional study design, and un-matched patient subgroups. These methodological issues currently hamper structural neuroimaging’s contribution to the diagnostic assessment of ADHD. Thus, standard clinical approaches to medication adjustment remain the best course when treatment ceases to provide positive effects (McGough, 2014; Barkley, 2015). Essential to good practice, clinicians must carefully document target symptoms, treatment responses, prescription information, dosage, and quantity of medication with each prescription, while also considering the possibility of abuse, misuse, and diversion of these drugs as the patient ages (McGough and McCracken, 2000; Craig et al., 2015; Rajeh et al., 2017). Patients with ADHD are prescribed about 3.8 times more amphetamine in 2005–2014 than methylphenidate (1.6 times from 2005 to 2014) (Moran et al., 2019). Moran et al. (2019) found that family medicine or internal medicine physicians (the most frequent prescribers) prescribed amphetamine about 72.5% of patients compared to pediatricians (51.6%) and psychiatrists (63.7%). This coincided with a rise of psychotic episodes based on prescription of these stimulants to ADHD cohorts (Moran et al., 2019). New onset psychosis was twice as high in ADHD patients that began amphetamine than among ADHD patients who began methylphenidate (Salo et al., 2013; Moran et al., 2019). Recall that methylphenidate acts as an inhibitor of dopamine transporters, whereas amphetamine releases four times as much dopamine in the synapse (Schiffer et al., 2006). The large heterogeneity of ADHD phenotypes also makes it difficult to access or appreciate the differences in catecholamine profiles. Medication selection should also be carefully considered based on comorbidities, co-medications, and pharmacokinetics such as patients who poorly metabolize pharmacological treatments (Bolea-Alamañac et al., 2014). This evidence suggests the need to cautiously screen for risk factors and to consistently monitor for reliability of the stimulant drug treatments after its administration to patients.

Neuroimaging studies have suggested that children with ADHD have diminished brain volume in certain subcortical structures compared to healthy controls (Castellanos et al., 2002; Qiu et al., 2009; Frodl and Skokauskas, 2012; Hoogman et al., 2017). Recently, a cross-sectional mega-analysis across the lifespan revealed that ADHD in late adulthood may have a delay in brain degeneration. For example, in healthy individuals the volume of the hippocampus and the amygdala normally declines later in life (Hoogman et al., 2017), and this decline is more pronounced in people with prodomal dementia (or mild cognitive impairment) and AD (Nho et al., 2012; Fujishima et al., 2014; Kehoe et al., 2014; Matsuda, 2016; Femminella et al., 2018). Interestingly, an ADHD diagnosis is associated with reduced volumetric decline in these structures later in life. It is important, however, to consider that these results were not influenced by psychostimulant use and no differences in volumes were found when comparing ADHD subjects who had never taken medication (82 patients) and ADHD subjects who used stimulant medication for more than 4 weeks (637 patients) (Hoogman et al., 2017). Our review summarizes that pharmaceutical treatment either leads to no changes or normalizes brain alterations in select brain regions in ADHD as quantified across a variety of neuroimaging modalities ranging from PET to MRI. While many studies have demonstrated decrease in volume and morphology attenuations following medication treatment (Semrud-Clikeman et al., 2006; Bledsoe et al., 2009; Ivanov et al., 2010; Sobel et al., 2010; van Elst et al., 2016; Douglas et al., 2018; Dutta, 2020), others did not find any measurable or quantifiable association of taking pharmaceutical drugs for ADHD on the brain. If ADHD poses a delay in brain degeneration later in life and if pharmaceutical treatment eliminates this neuroprotective element by normalizing structural changes associated with an ADHD diagnosis, then this benefit may be counteractive to neuroprotective volume into the geriatric years. On the other hand, pharmacological drugs for ADHD may potentially lead to neurodegenerative diseases. It is presumed that about 23% of cases with childhood ADHD will eventually develop MCI or dementia in older age, comparable to 21.5% of healthy subjects with no history of ADHD (Callahan et al., 2017). Whether there is a relationship between stimulant use during childhood or adult years from ADHD subjects and later MCI is largely unknown. It is important to note that screening methods for ADHD are often overlooked or confused with MCI diagnosis (Ivanchak et al., 2012), and there is a high frequency of antecedent ADHD symptoms to patients with dementia or AD (Ivanchak et al., 2012; Zhang et al., 2015; Fluegge and Fluegge, 2018). On the other hand, several lines of research in genetics (The Brainstorm Consortium et al., 2018; Pagoni et al., 2020) and neuroanatomy (Callahan et al., 2017) do not point to a direct link between ADHD and later MCI. There may be unrelated mediators that increase risk for later MCI or dementia, such as comorbidity (i.e., depression, anxiety, substance use) (Ivanchak et al., 2012) or stimulant use. Few neuroimaging and pharmacological studies have derived such relations as of yet, providing future opportunities to explore. Given that the evidence is inconclusive, it may be beneficial to act in a cautionary fashion when prescribing pharmaceutical treatment to ADHD. In this sense, one should weigh the benefits and limitations of medication treatment, including the severity of the disease presentation and comorbidities, against the potential risk of eliminating a benefit later in life.

The findings in this review paper have their limitations and their interpretations merit great caution. We did not use established methods of systematic review protocols and risk of bias (Higgins et al., 2011, 2019; Moher et al., 2015; Zeng et al., 2015), instead we tried to leverage a large number of existing and relevant studies. Therefore, the review may be flawed by selection bias, performance bias, detection bias, attrition bias, reporting bias, and other bias (Higgins et al., 2011). Our review may have missed studies due to bias in eligibility criteria and coverage of the retrieved studies. Additionally, similar to other reviews and meta-analytic studies (Cunill et al., 2016; Cortese et al., 2018), there are methodological and clinical heterogeneity in the included studies, such as study design, patient samples, and outcome measurements. It should be noted that our review does not support any pharmacological treatment over another and does not reveal any significant differences between the variations of short- and long- acting formulations. Few head-to-head studies exist due to insufficient patient samples and events that can be conducted to account for differences in dosage and intensity of all types of pharmacological treatments. Risk of bias in other studies is also important and can be more apparent than what was reported in our review. Therefore, there is still room for improvement in future systematic reviews regarding the topic of lifespan considerations for the pharmacological treatment of adult and pediatric ADHD. It is a limitation that only few databases were used in this review paper, of which it is likely that important studies were not captured.

In summary, subjects with ADHD may present differential trajectories of brain structure across the lifespan, with early abnormalities followed by a delay in brain degeneration later in life relative to controls. Drug treatment for ADHD does not always appear to increase global brain volume or attenuate morphology abnormalities across all white and gray matter. Rather, treatment seems to increase brain volume in a region-specific manner that normalizes ADHD brain structure, though these effects may vary throughout the lifespan. Indeed, additional studies are needed to determine the extent to which an ADHD diagnosis may be neuroprotective later in life. If so, determining the mechanisms that subtend this neuroprotective effect is essential. For example, it is reasonable to suggest that ADHD individuals switch more rapidly between stimuli to which they attend either externally (in hyperactive) or internally (in inattentive presentations). In this sense, the switching may in some sense exercise the brain. The delay in maturation hypothesis has continued to be a predominant theory in the field. Future studies may therefore focus on linking this maturation delay with delay in degeneration later in life. Unsupervised techniques (e.g., non-negative matrix factorization) (Anderson et al., 2014) may also be useful for identifying subgroups within or across the presentation domains that preferentially respond to pharmaceutical treatment throughout life, and the extent to which these subgroups overlap with those who may receive benefit from the behavioral manifestations of ADHD. Does the potential benefit of pharmaceutical treatment early in life (i.e., children, adolescents) outweigh the possibility that pharmaceutical treatment early in life may have detrimental effects in later adulthood? These questions must be examined in future longitudinal work in order to minimize the risk and maximize the utility of currently available ADHD medications.

## Author contributions

All authors listed have made a substantial, direct, and intellectual contribution to the work, and approved it for publication.
