# Supplementary material for: Neuroprotection in late life attention-deficit/hyperactivity disorder: A review of pharmacotherapy and phenotype across the lifespan
Source: Front Hum Neurosci. 2022 Sep 26;16:938501. doi: 10.3389/fnhum.2022.938501 (PMC9548548; doi:10.3389/fnhum.2022.938501)
Supplement: Supplementary file 1 [file Data_Sheet_1.doc]

**Supplementary Material**

We initialized our review process via a search on PubMed to find recent reviews and meta-analyses on ADHD, its pharmacotherapy, diagnosis, lifespan trajectory, neuroimaging and genetic correlates, and computational models of attention. These papers and the original research pertinent to our review at the intersection of these domains were included in this manuscript. This process also helped guide our formal review process by identifying relevant keywords for our formal search.

To identify additional sources, we conducted an additional formal systematic literature review from a broad range of electronic sources, including PubMed, EMBASE, PsycINFO, and Web of Science, having language (English) from published peer-review articles. A total of 4 main searches were done with the same databases with the following word strings. One was related to mechanisms of action for ADHD drugs with the following word strings: (methylphenidate OR amphetamine OR guanfacine OR psychostimulants OR non-stimulants OR alpha 2 adrenergic agonists OR medicated OR medication naive OR dopamine OR noradrenaline OR norepinephrine OR catecholamine) AND (ADHD OR attention deficit hyperactivity disorder OR hyperkinetic syndrome OR ADH) AND (children OR child OR adolescent OR pediatric OR adult OR adults). The second was related to neuroimaging studies of ADHD: (structural MRI OR neuroimaging OR PET OR positron emission tomography OR magnetic resonance imaging OR DTI OR diffusion tensor imaging) AND (ADHD OR attention deficit hyperactivity disorder OR hyperkinetic syndrome OR ADH) AND (children OR child OR adolescent OR pediatric OR adult OR adults). The third search was related to neurodevelopment, neurodegeneration, and ADHD neuroimaging relationships: (Alzheimer’s OR MCI OR mild cognitive disorder OR neurodegeneration) AND (neuroimaging OR magnetic resonance imaging OR MRI) AND (ADHD OR attention deficit hyperactivity disorder OR hyperkinetic syndrome OR ADH) AND (neurodevelopment OR lifespan). The final search was related to catecholamine neurocomputational models of attention: (ADHD OR attention deficit hyperactivity disorder OR hyperkinetic syndrome OR ADH) AND (dopamine OR noradrenaline OR noradrenaline OR catecholamine) AND (neurocomputational theories OR neurocomputational). Publications were excluded if it did not focus on neuroimaging dynamics in ADHD, relationships between neurodegeneration and ADHD or neurodevelopment and ADHD, catecholamine and attentional mechanisms from neurocomputational models, mechanisms of action of ADHD drugs or pharmacological effects of ADHD drugs. Additional articles were obtained via assessment of relevant papers through the search of references from articles and the authors knowledge. The search yielded 856 articles; of these, only 231 were considered relevant to the topic of interest.
